# Biocontrol of Soil-Borne Pathogens of *Solanum lycopersicum* L. and *Daucus* *carota* L. by Plant Growth-Promoting Actinomycetes: In Vitro and In Planta Antagonistic Activity

**DOI:** 10.3390/pathogens10101305

**Published:** 2021-10-12

**Authors:** Rihab Djebaili, Marika Pellegrini, Claudia Ercole, Beatrice Farda, Mahmoud Kitouni, Maddalena Del Gallo

**Affiliations:** 1 Department of Life, Health and Environmental Sciences, University of L’Aquila, Coppito, 67100 L’Aquila, Italy; djebaili.rihab@umc.edu.dz (R.D.); claudia.ercole@univaq.it (C.E.); beatrice.farda@student.univaq.it (B.F.); maddalena.delgallo@univaq.it (M.D.G.); 2 Laboratory of Microbiological Engineering and Applications, University of Brothers Mentouri Constantine 1, Ain El Bey Road, Constantine 25000, Algeria; mahmoudkitouni@yahoo.fr

**Keywords:** PGPB, actinomycetes, antifungal activity, antibacterial activity, cell culture filtrate, SEM, tomato, carrot

## Abstract

Biotic stress caused by pathogenic microorganisms leads to damage in crops. Tomato and carrot are among the most important vegetables cultivated worldwide. These plants are attacked by several pathogens, affecting their growth and productivity. Fourteen plant growth-promoting actinomycetes (PGPA) were screened for their in vitro biocontrol activity against *Solanum lycopersicum* and *Daucus carota* microbial phytopathogens. Their antifungal activity was evaluated against *Fusarium oxysporum* f. sp. *radicis-lycopersici* (FORL) and *Rhizoctonia solani* (RHS). Antibacterial activity was evaluated against *Pseudomonas syringae*, *Pseudomonas corrugata*, *Pseudomonas syringae* pv. *actinidiae*, and *Pectobacterium carotovorum* subsp. *carotovorum*. Strains that showed good in vitro results were further investigated in vitro (cell-free supernatants activity, scanning electron microscope observations of fungal inhibition). The consortium of the most active PGPA was then utilized as biocontrol agents in planta experiments on *S. lycopersicum* and *D. carota*. The *Streptomyces albidoflavus* H12 and *Nocardiopsis aegyptica* H14 strains showed the best in vitro biocontrol activities. The diffusible and volatile compounds and cell-free supernatants of these strains showed both antifungal (in vitro inhibition up to 85%, hyphal desegregation and fungicidal properties) and antibacterial activity (in vitro inhibition >25 mm and bactericidal properties). Their consortium was also able to counteract the infection symptoms of microbial phytopathogens during in planta experiments, improving plant status. The results obtained highlight the efficacy of the selected actinomycetes strains as biocontrol agents of *S. lycopersicum* and *D. carota*.

## 1. Introduction

Tomato (*Solanum lycopersicum* L.) and carrot (*Daucus carota* L.) are among the most important vegetables of the Solanaceae and Apiaceae families [1,2]. These plants are attacked by several soil-borne pathogens that induce severe diseases, which affect plant productivity. *Fusarium oxysporum* and *Rhizoctonia solani* are among the most important ubiquitous fungal pathogens of several plants, including tomato and carrot [3,4]. Several bacterial pathogens attacks can also affect tomato and carrot productivity. Among these, tomato spot disease caused by *Pseudomonas syringae* induces necrotic lesions on the different parts of the plant [5], and *Pectobacterium carotovorum* causes carrot soft rot [6]. Chemical treatments are available to counteract these diseases. However, these compounds are converted into toxic derivatives [7], and their application harms the environment and nontarget organisms [8]. Plants, through beneficial microorganism enlistment via root exudates, counteract the attacks of soil-borne pathogens [9,10]. Among these microorganisms, known as plant growth-promoting bacteria (PGPB), actinomycetes are an important source of bioactive and antimicrobial metabolites and control a wide range of phytopathogens [11]. Actinomycetes can be used as biostimulants, biopesticides, bioherbicides, and biological control agents [12,13], thanks to numerous agroactive compounds and through direct and indirect mechanisms [14]. The few effective formulations commercially available for agricultural use require an expansion of scientific knowledge on the subject. The present work aims to select a consortium of actinomycetes as a valid biocontrol agent for tomato and carrot. We screened the biocontrol capabilities of fourteen *Streptomyces* spp. and *Nocardiopsis* spp. actinomycetes that have already been demonstrated to be used as biostimulants [15] and salt stress-tolerance agents [16]. Given these capabilities, we hypothesized that these strains could be valid biocontrol agents against fungal and bacterial pathogens. To test our hypotheses, we screened the actinomycetes in vitro for antifungal and antibacterial antagonistic activities. We combined the most effective actinomycetes in a consortium and subjected them to further in vitro screening, evaluating the effects on fungal hyphae and the antibiofilm and antimicrobial activities of cell-free supernatants. Finally, we evaluated the efficacy of in planta biocontrol in tomato and carrot plants in greenhouse experiments.

## 2. Results

### 2.1. Antibacterial Activity

#### 2.1.1. In Vitro Antibacterial Activity of Selected Actinomycetes

The in vitro biocontrol activity against pathogenic bacteria was evaluated by dual culture, investigating the activity of diffusible compounds (classic Petri dishes). An interesting inhibitory effect was observed for seven of the fourteen actinomycetes. The other strains showed no inhibitory effect or moderate single-strain-specific inhibition (<25 mm). Table 1 presents the results recorded for actinomycetes with efficacy against at least two pathogenic strains.

*Streptomyces albidoflavus* H12 and *Nocardiopsis aegyptica* H14 showed the highest antibacterial activity in vitro, inhibiting all bacterial strains tested with an inhibition diameter >25 mm. Moderate inhibition (15–25 mm) against all the pathogenic strains was observed for *Streptomyces iakyrus* G10, *Streptomyces xantholiticus* G33, and *Streptomyces anulatus* J13, except for *P. syringae* (low inhibition, 5–15 mm). For *Streptomyces ambofaciens*, J27 was observed with only low antagonist activity against *Pc. carotovorum* strains, while for *N. aegyptica* S2, moderate inhibitory activity was observed for one of the *Pc. carotovorum* strains (PC2) and *P. corrugata.*

#### 2.1.2. Cell-Free Supernatants Minimum Inhibitory Concentration (MIC) and Maximum Bactericidal Concentration (MBC)

*S. albidoflavus* H12 and *N. aegyptica* H14 cell-free supernatants (CFSs) were investigated for their minimum inhibitory concentration (MIC) and minimum bactericidal concentration (MBC). The MIC and MBC values of the CFSs singularly and in combination are shown in Table 2.

The CFS of *S. albidoflavus* H12 was active at small concentrations only against the strain PC2 *Pc. carotovorum* (0.2–0.8%) with bactericidal activity (same values for MIC and MBC). Against *P. syringae* and *P. corrugata*, the MIC of this CFS was 25%. At this concentration, the activity was only bacteriostatic; bactericidal activities were obtained with 50% CFS. The observed activity for CFS of *Nocardiopsis aegyptica* H14 was slightly lower, with MIC values of 1.6% against *Pc. carotovorum* PC2 and 50% against *P. syringae* and *P. corrugata*. Bacteriostatic and bactericidal activity were observed at these concentrations, respectively. The combination of CFSs of the two strains did not affect the inhibitory and bactericidal activities observed for H12 and inhibitory and bacteriostatic activity against PC1 (MBC > 50%) was recorded.

#### 2.1.3. In Planta Biocontrol Activity

The growth parameters recorded in in planta experiments of tomato and carrot are shown in Table 3. Tomato and carrot plants treated with only actinomycetes consortium (CONS) showed increased survival and growth with respect to the control (CTL). The presence of pathogenic bacteria *P. syringae* and *P. corrugata* in tomato and *Pc. carotovorum* strains in carrot induced different negative effects on plants.

In tomato, in the presence of the pathogenic *P. syringae* (PS) infection alone, there was a decrease in survival in the number of leaves, in the length of root and shoot, and in the presence of damage on plants (*p <* 0.05). The simultaneous presence of the actinomycetes consortium with the bacterial pathogen (CONS + PS) improved plant response toward pathogenic attack, decreasing the damage and improving plant development (*p <* 0.05). *P. corrugata* (PC) pathogenesis affected survival and aerial parts of plants, damaging leaves and reducing shoot length and chlorophyll content compared to the control (CTL, *p <* 0.05). The simultaneous presence of the actinomycetes consortium (CONS + PC) protected the plants from these negative effects, improving their survival and developmental status (*p <* 0.05).

In carrot, infection of plants with both *Pc. carotovorum* strains (PC1 and PC2) induced damage and lower survival and chlorophyll content than the control (CTL, *p <* 0.05). Cotreatment with the actinomycetes consortium (CON S +PC1/PC2) reduced pathogenic negative effects, improving plant development compared to control in terms of root and shoots lengths and chlorophyll content (*p <* 0.05).

### 2.2. Antifungal Activity

#### 2.2.1. In Vitro Antifungal Activity of Selected Actinomycetes

**Figure 1:**
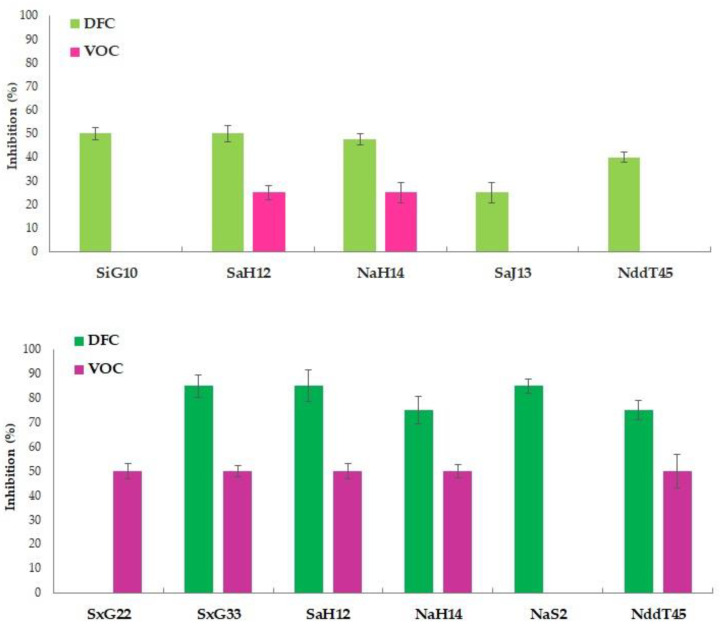
In vitro antifungal activity with diffusible compounds (DFCs) and volatile compounds VOCs of the selected actinomycetes strains against *Fusarium oxysporum* f. sp. *radicis-lycopersici* (graph on the top) and *Rhizoctonia solani* (graph on the bottom). In the figure: SiG10, *Streptomyces iakyrus* G10; SaH12, *Streptomyces albidoflavus* H12; NaH14, *Nocardiopsis aegyptica* H14; SaJ13, *Streptomyces anulatus* J13; NddT45, *Nocardiopsis dassonvillei* subsp. *dassonvillei* T45; SxG22, *Strepptomyces xantholiticus* G22; SxG33, *Strepptomyces xantholiticus* G33; NaS2, *N. aegyptica* S2.

The results of in vitro antifungal activity of actinomycetes with interesting inhibition percentages (>25%) are presented in Figure 1.

In vitro mycelium growth of *F. oxysporum* f. sp. *radicis-lycopersici* was inhibited at ~50% by diffusible compounds of *S. albidoflavus* H12 and *N. aegyptica* H14. For these two strains, the mycelial inhibition was also obtained from volatile compounds, with percentages of 25%. Among the other strains, an interesting inhibition was recorded for the diffusible compounds of *S. iakyrus* G10 (50%), *S. anulatus* J13 (25%), and *N. dassonvillei* subsp. *dassonvillei* T45 (35%). The in vitro growth of the mycelium of *R. solani* was inhibited by a greater number of actinomycetes and with higher rates of inhibition. Mycelium inhibitions greater than 70% were recorded by diffusible compounds of *S. xantholiticus* G33, *S. albidoflavus* H12, *N. aegyptica* H14, *N. aegyptica* S2, and *N. dassonvillei* subsp. *dassonvillei* T45. With the exception of *N. aegyptica* S2, the volatile compounds of all these strains, together with those of *S. xantholiticus* G22, were able to inhibit the growth of *R. solani* by 50%. Given the best performances, the antifungal abilities of *S. albidoflavus* H12 and *N. aegyptica* H14 were further investigated.

#### 2.2.2. Actinomycetes Effects on the Hyphal Structure

**Figure 2:**
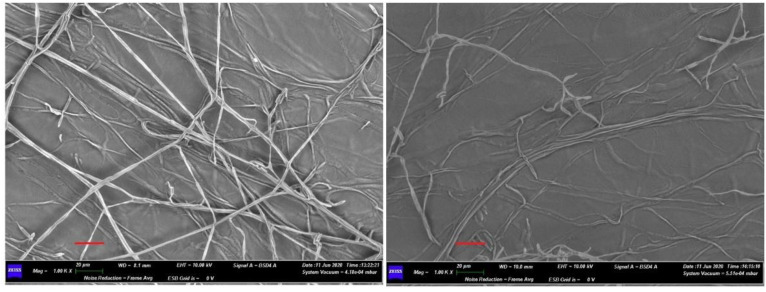
SEM micrographs of *Fusarium oxysporum* f. sp. *radicis-lycopersici* hyphae in the absence (on the left) and in the presence (on the right) of actinomycetes. The panel on the left shows the normal branching of hyphae. In the right panel, thinner and more distorted hyphal structures can be seen, with many disaggregation points. Scale bars (in red) 20 µm.

**Figure 3:**
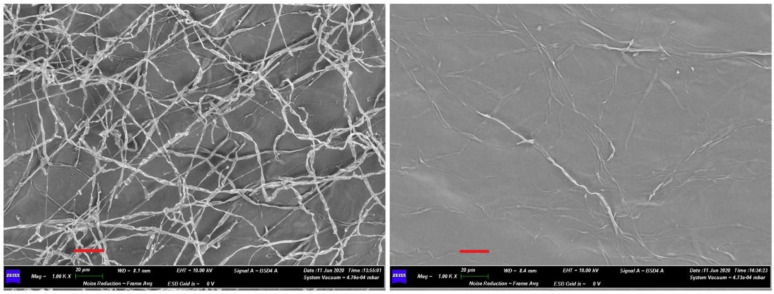
SEM micrographs of *Rhizoctonia solani* hyphae in the absence (on the left) and in the presence (on the right) of actinomycetes. The panel on the left shows the normal hyphal structure and development. In the panel on the right, the inhibition of development by disruption of the hyphal structures can be observed. Scale bars (in red) 20 µm.

The effects of *Streptomyces albidoflavus* H12 and *N. aegyptica* H14 on fungal mycelia were investigated by scanning electron microscopy (SEM). Micrographs of FORL and RHS mycelia in the absence and presence of actinomycetes are presented in Figure 2 and Figure 3, respectively.

Micrographs of FORL and RHS mycelia grown without actinomycetes (left panels of Figure 2 and Figure 3) showed branched and intertwined hyphae, with continuous structures. The presence of actinomycetes (right panels in Figure 2 and Figure 3) induced morphological deterioration of the hyphae and a change in structures, which appear discontinuous and distorted.

#### 2.2.3. Cell-Free Supernatants Minimum Inhibitory Concentration (MIC) and Minimum Fungicidal Concentration (MFC)

CFS of *S. albidoflavus* H12 and *N. aegyptica* H14 were assayed for MIC and MFC against *F. oxysporum* f. sp. *radicis-lycopersici* and *Rhizoctonia solani.* The results of CFS tested individually and in combination are presented in Table 4.

Active MICs against *F. oxysporum* f. sp. *radicis-lycopersici* ranged from 0.4 to 0.8%, with greater inhibition by CFS of *N. aegyptica* H14. All these concentrations had fungicidal properties (MIC = MFC). For *R. solani*, the MIC and MFC values of *N. aegyptica* H14 (0.2% and 0.4%) were lower than those of *S. albidoflavus* H12 (0.8%). However, for both fungal pathogens, when the CFSs of the two actinomycetes were pooled, fungicidal activities comparable to the lowest values observed for *N. aegyptica* H14 were observed (0.4%).

#### 2.2.4. In Planta Biocontrol Activity

As found during the in planta antibacterial activity experiments (Table 5), the survival and growth of tomato and carrot plants treated with the actinomycetes consortium (CONS) alone were higher than the control (CTL). The presence of pathogenic bacteria *P. syringae* and *P. corrugata* in tomato and *Pc. carotovorum* strains in carrot induced several negative effects on plants.

In tomato, *F. oxysporum* f. sp. *radicis-lycopersici* infection (FORL) reduced survival, caused severe damage, and negatively affected plant shoot and root development. Cotreatment of plants with fungal infection and actinomycetes consortium (CONS + FORL) improved plant status, allowing normal growth and improving some development parameters. Compared to the control (CTL), plants with consortium inoculation and pathogenic infection (CONS + FORL) had lower survival and shoot length (*p <* 0.05). Nonetheless, a similar number of leaves (*p >* 0.05) and higher chlorophyll content and root length (*p <* 0.05) were shown. A similar situation was observed for *R. solani.* The pathogenic attack of this fungus (RHS) reduced the survival and growth of plants. In the copresence of actinomycetes consortium (CONS + RHS), the pathogenesis was still evident. Compared to control, decreases in growth and development were observed (*p <* 0.05). However, the attack was less offensive, and less damage and fewer negative effects on plant development than RHS were observed (*p <* 0.05).

The *R. solani* pathogenesis on carrot (RHS) strongly reduced plant survival and all developmental parameters. The simultaneous presence of the actinomycetes consortium (CONS + RHS), even if not completely, significantly reduced the negative effects on survival; compared to the control, similar root length, amount of chlorophyll (CTL, *p >* 0.05), and higher shoot length and number of leaves (*p* < 0.05) were observed.

**Figure 4:**
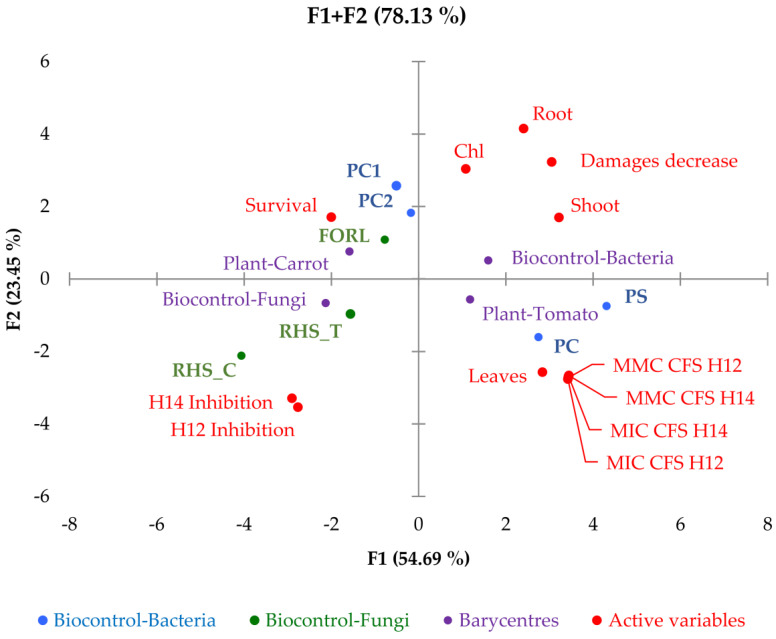
Biplot (scores and loadings) obtained from the principal component analysis on a dataset of in vitro and in planta results (increase in the percentages in plants treated with the actinomycetes consortium and infected with pathogens compared to plants with the only infection). The tested plant and fungal or bacterial biocontrol were considered as supplementary categorical variables. In the Figure: PS, *Pseudomonas syringae*; PC, *Pseudomonas corrugata,* PC1/PC2, *Pectobacterium carotovorum* strain 1 and strain 2; FORL, *Fusarium oxysporum* f. sp. *radicis-lycopersici;* RHS, *Rhizoctonia solani.* Chl, chlorophyll; H12/H14 Inhibition, in vitro bacterial and fungal inhibition by *Streptomyces albidoflavus* H12/*Nocardiopsis aegyptica* H14; MIC CFS H12/H14, minimum inhibitory concentrations of CFS of *S. albidoflavus* H12/*N. aegyptica* H14; MMC CFS H12/H14, minimum microbicidal concentrations of CFS of *S. albidoflavus* H12/*N. aegyptica* H14.

To investigate the contribution of the in vitro antimicrobial properties of *S. albidoflavus* H12 and *N. aegyptica* H14 on bacterial and fungal biocontrol in planta, the results were included in a single dataset and processed by a principal component analysis. The biplot is presented in Figure 4.

The first principal component (F1) accounted for a total variance of 55% and the second (F2) of 24%; the total variance explained by these two components was 78%. The biplot grouping, based on data correlations, showed a correlation of in planta fungal biocontrol of tomato and carrot with the in vitro inhibitory activities shown for *S. albidoflavus* H12 and *N. aegyptica* H14. Experimental conditions in planta of carrot bacterial pathogens were included in this grouping, together with an increased survival rate in plants treated with actinomycetes consortium and infected with pathogens, compared to plants with the sole infection. The other parameters in planta had the same correlations of bacterial biocontrol, with the exception of leaves number, which had similar correlations to in planta experimental conditions of tomato bacterial pathogens and to the in vitro inhibitory and microbicidal concentrations found for CFS *S. albidoflavus* H12 and *N. aegyptica* H14. These correlations underline that, at a statistical level, the differences in planta shown between plants treated with actinomycetes consortium and infected with pathogens and plants with the sole infection can be ascribed to in vitro properties highlighted by different tests. The fungal biocontrol was more correlated (and thus ascribed) to the in vitro inhibitory activities shown for *S. albidoflavus* H12 and *N. aegyptica* H14. Bacterial biocontrol was more correlated/ascribed to the activity of the metabolites present in the CFS of *S. albidoflavus* H12 and *N. aegyptica* H14.

## 3. Discussion

Diseases caused by pathogenic bacteria and fungi cause significant damage to crops, resulting in decreased yields and increased costs. To address this problem, farmers usually use synthetic chemicals, with major negative consequences for the environment and human health. The present work focused on the evaluation of several beneficial actinomycetes as biocontrol agents against bacterial and fungal pathogenic strains of tomato and carrot. Our approach made it possible to select a valid consortium useful for combating the selected pathogens. The in vitro assays and in planta experiments highlighted the suitability of the consortium formed by *Streptomyces albidoflavus* H12 and *Nocardiopsis aegyptica* H14 as a biocontrol agent. The consortium was able to control in planta bacterial and fungal pathogenic attacks. The biocontrol activity could be ascribed to the different abilities of *S. albidoflavus* H12 and *N. aegyptica* H14. This study also highlighted a contribution of the antibacterial and antifungal metabolites present in cell-free supernatants of both strains with bactericidal and fungicidal properties.

The antibacterial and antifungal activities are common traits found among actinomycetes strains [11,17,18,19,20,21,22,23,24,25]. The biocontrol properties of *Streptomyces* and *Nocardiopsis* genera are mainly related to the release of extracellular metabolites, siderophores, and enzymes, with a broad antimicrobial spectrum [25,26,27]. The genus *Streptomyces* is the major producer of antibiotics. It plays an important role in the suppression of plant diseases induced by bacterial pathogens and has a great diversity of genes for the production of biocontrol secondary metabolites [28,29].

The biocontrol of bacterial plant pathogens by *Streptomyces* has been reported in many studies [17,30,31]. Diffusible and volatile antibacterial metabolites have direct activity on pathogenic bacterial cells and on their *quorum sensing*, disrupting the gene expression regulation in response to cell-population density and biofilm formation [32]. The ability of *Streptomyces* to control plant diseases caused by fungal pathogens is widely described for *F. oxysporum* [20,33,34,35,36,37] and *R. solani* [7,38,39,40,41,42,43]. The diffusible and volatile compounds produced by *Streptomyces* also act against the polymeric compounds of fungal pathogens, such as chitin [44]. Cell-free supernatants produced by *Streptomyces* are a valid source of biocontrol metabolites against *F. oxysporum* and *R. solani* [45,46,47,48,49]. Recent work by Lyu and collaborators demonstrated that reveromycin A and B—polyketides compounds with strong antifungal activity—extracted from *Streptomyces* sp. 3–10 CFS inhibit several phytopathogenic fungi, including *R. solani* [50].

The genus *Nocardiopsis* produces a large variety of bioactive compounds, including antimicrobial agents and enzymes [51]. The biocontrol compounds described for this genus are pendolmycin, a strong antifungal agent, and griseusins, thiopeptides, and naphthospironones, with antimicrobial power [51]. The activity against bacterial and fungal plant pathogens is less reported than in *Streptomyces.* However, metabolites of this genus have been reported to be effective antimicrobial and antifungal agents [52]. The recent work by Sabu and collaborators described a good biocontrol attitude of a *Nocardiopsis* sp. isolate, with broad antibacterial, antibiofilm, and antiphytopathogenic activity [53]. The CFS compounds of a halophilic *Nocardiopsis gilva* isolate have been reported to contain a novel p-terphenyl 1 compound with in vitro inhibitory activities against *Fusarium* spp. [54].

The in planta biocontrol was tested by combining the most efficient isolates; this strategy was in line with literature evidence supporting the use of microbial consortia for the control of microbial phytopathogens [8,55,56,57,58]. The efficacy of in planta biocontrol shown by the consortium of *S. albidoflavus* H12 and *N. aegyptica* H14 can be ascribed not only to the activities mentioned above, but also to other indirect mechanisms. Once associated with plant rhizosphere and tissues, actinomycetes counteract microbial phytopathogens by promoting plant growth and development through the solubilization of phosphates, indoleacetic acid, hydrogen cyanide, and ammonia production [59]. Actinomycetes also induce systemic resistance [60], promoting a faster and stronger response of plants to pathogenic attacks [61].

## 4. Materials and Methods

The following fourteen strains of actinomycetes, previously isolated and characterized [62], were used in this study:

- *Nocardiopsis aegyptica* H14 (MG597543).
- *Nocardiopsis aegyptica* S2 (MG597572).
- *Nocardiopsis alba* J21 (MG597576).
- *Nocardiopsis dassonvillei* subsp. *dassonvillei* D14 (MG597514).
- *Nocardiopsis dassonvillei* subsp. *dassonvillei* T45 (MG597502).
- *Streptomyces albidoflavus* H12 (MG597552).
- *Streptomyces ambofaciens* J27 (MG597599).
- *Streptomyces anulatus* J13 (MG597579).
- *Streptomyces iakyrus* G10 (MG597593).
- *Streptomyces thinghirensis* K23 (MG597560).
- *Streptomyces thinghirensis* J4 (MG597590).
- *Streptomyces xantholiticus* K12 (MG597545).
- *Streptomyces xantholiticus* G22 (MG597582).
- *Streptomyces xantholiticus* G33 (MG597585).

Cultures of the strains were grown on International *Streptomyces* Project No. 2 (ISP2) medium at 30 °C for 7 d.

### 4.1. Antibacterial Activity

The fourteen isolates listed above were screened for in vitro biocontrol activity against pathogenic bacterial strains by the dual-culture method. Cell-free supernatants of the most effective strains (MIC up to 0.8%) were combined and assayed by minimum inhibitory concentration (MIC) and minimum bactericidal activity (MBC) on 96-well plates. Broth cultures of the most effective strains were also combined in a consortium and tested for in planta biocontrol effectiveness under greenhouse experiments. Phytopathogenic bacteria *Pseudomonas syringae* pv. *tomato* (PS) and *Pseudomonas corrugata* pv. *tomato* (PC)—from tomato—and *Pectobacterium carotovorum* subsp. *carotovorum* strains PC1 and PC2—from carrot—were provided by Prof. Giuliano Bonanomi of the Dept. of Agriculture, University of Naples Federico II (Italy).

#### 4.1.1. In Vitro Antibacterial Activity of Selected Actinomycetes

The in vitro antibacterial activity was evaluated by cocultivation of bacterial pathogenic strains with agar plugs of actinomycetes. A 100 µL volume of nutrient broth (NB) cultures of pathogenic strains, grown overnight at 37° under 150 rpm with constant shaking, was spread on the plate of ISP2/NA (nutrient agar) dishes (Ø 90 mm dishes, 50% ISP2 and 50% NA). Agar plugs of 5 d old selected actinomycetes strains grown on ISP2 solid medium were placed on streaked ISP2/NA plates, and dishes were incubated at 37 °C for 24 h. The inhibition zones formed around the agar plugs after cultivation were measured with a ruler.

#### 4.1.2. Cell-Free Supernatants Minimum Inhibitory Concentration (MIC) and Minimum Bactericidal Concentration (MBC)

*Streptomyces albidoflavus* H12 and *Nocardiopsis aegyptica* H14 cell-free supernatants (CFSs) were investigated for antibacterial activity following the CLSI guidelines [63]. CFSs were obtained from broth cultures of ISP2 strains grown at 30 °C for 7 d with constant agitation (150 rpm). Cultures were centrifuged at 10,000 *g* for 10 min and filtered through a 0.22 μm bacteriological filter. CFSs were tested singularly and in combination (50:50). Round-bottom 96-well plates and nutrient broth (NB) medium were used for the test, and the readings were carried out spectrophotometrically, after incubation at 37 °C for 24 h, by resazurin (Alamar blue) addition [64]. For each CFS, the minimum inhibitory concentration (MIC) was identified in the well without growth containing the lower CFS concentration. Minimum bactericidal concentration (MBC) was estimated by plating 100 µL of the wells in which there was no growth on NB medium. After incubation of the plates at 37 °C for 24 h, the corresponding concentration of CFS was considered bactericidal in the absence of colonies, while it was considered bacteriostatic in the presence of colonies.

#### 4.1.3. In Planta Antibacterial Activity

The consortium’s in planta antimicrobial activity was evaluated on tomato, against PS and PC strains, and on carrot, against PC1 and PC2. The experimental conditions were arranged as follows:

- CTL: control, untreated plants.
- CONS: plants inoculated only with actinomycetes.
- CONS + PS/PC/PC1/PC2: plants treated with actinomycetes and infected with a bacterial pathogen.
- PS/PC/PC1/PC2: plants infected only with a bacterial pathogen.

Plant seeds were treated with 10^8^ spore mL^−1^ from the actinomycetes consortium (equal amounts of *S. albidoflavus* H12 and *N. aegyptica* broth cultures) and allowed to dry overnight at room temperature. Each experimental condition comprised 25 pots with two seeds per pot (6/7 diameter) filled with common soil (pH = 8.2, electrical conductivity = 1 ds/m, total porosity = 81% (*v/v*)). Infections were realized after plant germination, using bacterial suspensions of 10^6^ CFU mL^−1^. The experiments were carried out under natural light and environmental temperature until the symptoms of the disease manifested. Induced protection was evaluated by estimating plant survival, number of leaves, shoot and root lengths, degree of damage, and chlorophyll content [2].

### 4.2. Antifungal Activity

The fourteen actinomycetes were screened for in vitro biocontrol activity against two fungal pathogens of tomato and carrot, *Rhizoctonia solani* and *Fusarium oxysporum* f. sp. *radicis-lycopersici* (provided by Prof. Giuliano Bonanomi of the Dept. of Agriculture, University of Naples Federico II, Italy). The in vitro biocontrol activity against fungal strains was assessed by dual culture, studying the inhibitory activities of diffusible (classic Petri dishes) and volatile (biplate Petri dishes) compounds. The inhibition zones of the most active strains were also studied by SEM. The cell-free supernatants of the most effective strains were combined and assayed by minimum inhibitory concentration (MIC) and minimal fungicidal concentration (MFC). Broth cultures of the most effective strains were also combined in a consortium and tested for efficacy in planta biocontrol.

#### 4.2.1. In Vitro Antifungal Activity of Selected Actinomycetes (Diffusible and Volatile Compounds)

The in vitro antagonistic activity was evaluated by cocultivation of fungal pathogens with actinomycetes strains. Inhibition of diffusible compounds was estimated by ISP2/potato dextrose agar (PDA) plates (50:50) using classic Petri dishes. Volatile compounds were estimated using biplate Petri dishes containing ISP2 for actinomycetes and PDA for fungi. Actinomycetes and fungi were transferred to plates using agar plugs (Ø 5 mm) obtained from 5 d cultures. For both plate types of fungi, positive controls were prepared without the addition of plugs. Dishes were incubated at 28 °C until the mycelium covered the entire plate of the positive fungi control (5–7 d). Each trial was repeated 3 times, and the inhibition percentages were calculated as follows:

$$I\;\%\;=\;\frac{\left(\mathrm{mm}\;\mathrm{growth}\;\mathrm{control}\;-\;\mathrm{mm}\;\mathrm{growth}\;\mathrm{dual}\;\mathrm{culture}\right)}{\mathrm{mm}\;\mathrm{growth}\;\mathrm{control}}\;\times \;100$$

#### 4.2.2. Actinomycetes Effects on the Hyphal Structure

The effects of actinomycetes strains on *F. oxysporum* f. sp. *radicis-lycopersici* (FORL) and *R. solani* (RHS) were investigated by scanning electron microscopy (SEM), using a Gemini SEM 500 SEM (Zeiss, Oberkochen, Germany). Small pieces of agar from the actinomycete/fungus interaction zone and the fungus growth control were taken and mounted on adhesive tape. Freshly collected samples were observed using a specific stage for Peltier technology, with a working temperature of −1 °C. Micrographs acquisition was performed with a working distance of 8.1 mm, an acceleration voltage of 10 kV, and using a backscattered electrons detector (BSD4 signal).

#### 4.2.3. Cell-Free Supernatants Minimum Inhibitory Concentration (MIC) and Minimum Fungicidal Concentration (MFC)

*Streptomyces albidoflavus* H12 and *Nocardiopsis aegyptica* H14 cell-free supernatants (CFSs) were also investigated for antifungal activity following the CLSI guidelines [64]. The CFSs were obtained as described in Section 4.1.2. CFSs were tested singularly and in combination (50:50) using 96-well round-bottom plates and PDB medium. The readings were performed spectrophotometrically, after incubation at 28 °C for 5 d, by adding resazurin (Alamar blue, Bio-Rad, Hercules, CA, USA) [64]. For each CFS, the minimum inhibitory concentration (MIC) was identified in the well without growth containing the lower CFS concentration. The minimum fungicidal concentration (MFC) was estimated by plating 100 µL of the wells in which there was no growth on PDA medium. After incubation of the plates at 28 °C for 5 d, the corresponding concentration of CFS was considered fungicidal in the absence of spore germination, while it was considered fungistatic in the presence of growth.

#### 4.2.4. In Planta Antifungal Activity

The consortium’s in planta antifungal activity was evaluated on tomato against FORL and RHS and on carrot against RHS. The experimental conditions were arranged as follows:

- CTL: control, untreated plants.
- CONS: plants inoculated only with actinomycetes.
- CONS + FORL/RHS: plants treated with actinomycetes and infected with a fungal pathogen.
- FORL/RHS: plants infected only with a fungal pathogen.

Treatment of seeds with actinomycetes was carried out as described in Section 4.1.3. Fungal infections were carried out by inoculating the plants with the fungal spore/mycelial suspensions as previously described [2]. Twenty-five pots with two seeds per pot (6/7 diameter) were arranged for each experimental condition. The pots were filled with common soil, and the experiments were carried out under natural light and environmental temperature. Once symptoms of the disease manifested, plant survival, number of leaves, shoot and root lengths, degree of damage, and chlorophyll content were estimated [2].

### 4.3. Statistical Analyses

Data were analyzed by one-way analysis of variance (ANOVA), utilizing Fisher’s least significant difference (LSD) post-hoc test to compare the mean values to a significance level of 5% (*p* < 0.05). The principal component analysis (PCA) algorithm was used to decompose the dataset of in vitro and in planta results (increase the percentages in plants treated with actinomycetes consortium and infected with pathogens respect to plants with only infection). The plant tested and fungal or bacterial biocontrol were considered as supplementary categorical variables (columns). In Figure 4: Ps, *Pseudomonas syringae*; Pc, *Pseudomonas corrugata,* PC1/PC2, *Pectobacterium carotovorum* strain 1 and strain 2; FORL, *Fusarium oxysporum* f. sp. *radicis-lycopersici;* RHS, *Rhizoctonia solani.* All statistical analyses were carried out using XLSTAT 2014 software (Addinsoft, Paris, France).

## 5. Conclusions

Currently, agriculture is largely dependent on agrochemicals, with a negative impact on ecosystems. Alternatives are needed such as the use of bioagents to fight plant diseases. In this study, several methods were used to determine the antagonistic activity of actinomycetes isolates against various pathogenic fungi and bacteria. The results obtained showed the in vitro power to inhibit pathogenic strains with the dual-culture method and with cell-free supernatants. The results demonstrated good in vitro potential against both bacterial and fungal pathogens. The most effective strains, *Streptomyces albidoflavus* H12 and *Nocardiopsis aegyptica* H14, were further investigated and grouped in a consortium for testing in in planta experiments. In general, for both carrot and tomato plants, treatment with the bacterial consortium counteracted microbial infections, improving growth and development.

The results of this work are encouraging. These strains have shown good biofertilization and salt stress control properties in previous studies. More in-depth studies are needed on the characterization of secondary metabolites, on plant association, and on the formulation of consortia. The suitability of applying these strains against other pathogens and in the open fields also requires further research. However, our results underline the possible role of these actinomycetes as biocontrol agents in the sustainable management of tomato and carrot crops.

## Figures and Tables

**Table 1:** In vitro antibacterial activity of the selected actinomycetes strains against *Pseudomonas syringae, Pseudomonas corrugata*, and *Pectobacterium carotovorum* subsp. *carotovorum* (PC1 and PC2).

	SiG10	SxG33	SaH12	NaH14	SaJ13	SaJ27	NaS2
*P. syringae*	+	+	+++	+++	+	−	−
*P. corrugata*	++	++	+++	+++	++	−	++
*Pc. carotovorum* PC1	++	++	+++	+++	++	+	−
*Pc. carotovorum* PC2	++	++	+++	+++	++	+	++

+++, high inhibition, > 25 mm; ++, moderate inhibition, (15–25 mm); + low inhibition, (5–15 mm); −, no inhibition. SiG10, *Streptomyces iakyrus* G10; SxG33, *Streptomyces xantholiticus* G33; SaH12, *Streptomyces albidoflavus* H12; NaH14, *Nocardiopsis aegyptica* H14; SaJ13, *Streptomyces anulatus* J13; SaJ27, *Streptomyces ambofaciens* J27; NaS2, *N. aegyptica* S2.

**Table 2:** Minimum inhibitory concentration (MIC) and minimum bactericidal concentration (MBC) percentages of *Streptomyces albidoflavus* H12 and *Nocardiopsis aegyptica* H14 cell-free supernatants (singularly and in combination) against *Pseudomonas syringae, Pseudomonas corrugata*, and *Pectobacterium carotovorum* subsp. *carotovorum* (PC1 and PC2).

Pathogenic Bacteria	SaH12 CFS		NaH14 CFS		SaH12 + NaH14 CFS	
	MIC	MBC	MIC	MBC	MIC	MBC
*P. syringae*	25.5	50.0	50.0	50.0	25.0	50.0
*P. corrugata*	25.5	50.0	50.0	50.0	25.0	50.0
*Pc. carotovorum* PC1	-	-	-	-	50.0	-
*Pc. carotovorum* PC2	0.8	0.8	0.8	1.6	0.8	0.8

In the table: SaH12, *Streptomyces albidoflavus* H12; NaH14, *Nocardiopsis aegyptica* H14; CFS, cell-free supernatants.

**Table 3:** In planta antibacterial activity of the consortium of *Streptomyces albidoflavus* H12 and *Nocardiopsis aegyptica* H14 against *Pseudomonas syringae* and *Pseudomonas corrugata* in tomato and *Pectobacterium carotovorum* strains in carrot.

	Survival (%)	Leaves (n°)	Damage (Grade)	Root (cm)	Shoot (cm)	Chlorophyll (mg g FW^−1^)
Tomato						
CTL	85.90 ^b^	3.40 ^c^	-	2.30 ^c^	7.04 ^c^	0.94 ^b^
CONS	98.80 ^a^	5.30 ^a^	-	3.99 ^a^	11.09 ^a^	1.08 ^a^
CONS + PS	64.70 ^c^	4.80 ^a,b^	2.80 ^c^	2.96 ^b^	9.32 ^b^	1.08 ^a^
CONS + PC	87.00 ^b^	4.60 ^b^	3.50 ^b^	2.79 ^b^	8.89 ^b^	1.00 ^b^
						
PS	60.70 ^e^	2.70 ^d^	2.50 ^a^	2.02 ^d^	6.06 ^e^	0.62 ^c^
PC	50.40 ^d^	2.20 ^d^	4.70 ^c^	1.25 ^c^	4.15 ^d^	0.62 ^c^
LSD	1.24	0.63	0.47	0.37	0.88	0.06
Carrot						
CTL	88.00 ^B^	2.00 ^B^	-	1.79 ^B^	2.60 ^D,E^	0.89 ^C^
CONS	98.60 ^A^	3.70 ^A^	-	3.35 ^A^	4.90 ^A^	1.10 ^A^
CONS + PC1	86.80 ^B^	2.00 ^B^	2.50 ^B^	3.08 ^A^	4.27 ^B^	1.08 ^A^
CONS + PC2	40.80 ^C^	2.00 ^B^	2.50 ^B^	3.07 ^A^	3.78 ^C^	0.99 ^B^
PC1	40.20 ^C^	2.00 ^B^	4.80 ^A^	1.40 ^B^	2.65 ^D^	0.61 ^D^
PC2	27.40 ^D^	2.00 ^B^	4.50 ^A^	1.63 ^B^	2.16 ^E^	0.61 ^D^
LSD	1.40	0.18	0.37	0.40	0.48	0.07

In the table: CONS, consortium formed by Streptomyces albidoflavus H12 and Nocardiopsis aegyptica H14; CTL, control; PS, *Pseudomonas syringae*; PC, *Pseudomonas corrugata*; PC1, *Pectobacterium carotovorum* subsp. *carotovorum* strain 1; PC2, *Pectobacterium carotovorum* subsp. *carotovorum* strain 2; LSD, least significant difference. Results followed by the same case letter are not significantly different according to Fisher’s LSD post-hoc test. Lowercase letters (a–d) refer to the comparison of tomato experimental conditions. Uppercase letters (A–E) refer to the comparison of carrot experimental conditions.

**Table 4:** Minimum inhibitory concentration (MIC) and minimum fungicidal concentration (MFC) percentages of *Streptomyces albidoflavus* H12 and *Nocardiopsis aegyptica* H14 cell-free supernatants (singularly and in combination) against *Rhizoctonia solani* and *Fusarium oxysporum* f. sp. *radicis-lycopersici*.

Pathogenic Bacteria	SaH12 CFS		NaH14 CFS		SaH12 + NaH14 CFS	
	MIC	MFC	MIC	MFC	MIC	MFC
*Fusarium oxysporum*	0.8	0.8	0.4	0.4	0.4	0.4
*Rhizoctonia solani*	0.8	0.8	0.2	0.4	0.4	0.4

In the table: SaH12, *Streptomyces albidoflavus* H12; NeH14, *Nocardiopsis aegyptica* H14; CFS, cell-free supernatants.

**Table 5:** In planta antifungal activity of *Streptomyces albidoflavus* H12 and *Nocardiopsis aegyptica* H14 consortium against *Fusarium oxysporum* f. sp. *radicis-lycopersici* and *Rhizoctonia solani* in tomato and *R. solani* in carrot.

	Survival (%)	Leaves (n°)	Damage (Grade)	Root (cm)	Shoot (cm)	Chlorophyll (mg g FW^−1^)
Tomato						
CONS	98.40 ^a^	6.00 ^a^	-	3.85 ^a^	10.71 ^a^	1.10 ^a^
CTL	86.40 ^b^	3.60 ^b,c^	-	2.98 ^c^	7.71 ^b^	0.94 ^b^
CONS + FORL	78.50 ^c^	4.00 ^b^	2.60 ^b^	3.51 ^b^	6.15 ^c^	1.07 ^a^
CONS + RHS	60.30 ^d^	3.40 ^c^	2.60 ^b^	3.37 ^b^	5.90 ^c^	0.72 ^c^
FORL	53.60 ^e^	2.60 ^d^	4.60 ^a^	1.76 ^e^	4.69 ^d^	0.39 ^d^
RHS	49.60 ^f^	2.00 ^e^	4.60 ^a^	2.23 ^d^	3.93 ^e^	0.42 ^d^
LSD	1.30	0.56	0.38	0.25	0.51	0.09
Carrot						
CONS	98.70 ^A^	3.70 ^A^	-	3.10 ^A^	4.89 ^A^	0.84 ^A^
CTL	88.00 ^B^	2.00 ^C^	-	2.30 ^B^	3.20 ^B^	0.94 ^A^
CONS + RHS	44.30 ^C^	2.50 ^B^	2.70 ^B^	2.40 ^B^	4.80 ^A^	1.06 ^A^
RHS	20.20 ^D^	1.50 ^D^	4.80 ^A^	1.43 ^C^	2.60 ^C^	0.57 ^B^
LSD	2.13	0.40	0.36	0.28	0.52	0.24

In the table: CONS, consortium formed by *Streptomyces albidoflavus* H12 and *Nocardiopsis aegyptica* H14; CTL, control; FORL, *Fusarium oxysporum* f. sp. *radicis-lycopersici*; RHS, *Rhizoctonia solani*; LSD, least significant difference. Results followed by the same case letter are not significantly different according to Fisher’s LSD post-hoc test. Lowercase letters (a–e) refer to the comparison of tomato experimental conditions. Uppercase letters (A–D) refer to the comparison of carrot experimental conditions.

## Data Availability

The data that support the findings of this study are available upon request from the corresponding author.
